# Microbial Dynamics in Ophthalmic Health: Exploring the Interplay between Human Microbiota and Glaucoma Pathogenesis

**DOI:** 10.3390/medicina60040592

**Published:** 2024-04-03

**Authors:** Joicye Hernández-Zulueta, Andres J. Bolaños-Chang, Francisco J. Santa Cruz-Pavlovich, América D. Valero Rodríguez, Alejandro Lizárraga Madrigal, Ximena I. Del Rio-Murillo, José Navarro-Partida, Alejandro Gonzalez-De la Rosa

**Affiliations:** 1 Departamento de Biología Celular y Molecular, Centro Universitario de Ciencias Biológicas y Agropecuarias, Universidad de Guadalajara, Av. Ing. Ramón Padilla Sánchez, Zapopan 45200, Jalisco, Mexico; 2 Tecnologico de Monterrey, Escuela de Medicina y Ciencias de la Salud, Monterrey 64849, Nuevo Leon, Mexico; 3 Centro de Retina Medica y Quirúrgica, S.C., Hospital Puerta de Hierro, Zapopan 45116, Jalisco, Mexico

**Keywords:** microbiome, glaucoma, ocular homeostasis, dysbiosis

## Abstract

The human microbiome has a crucial role in the homeostasis and health of the host. These microorganisms along with their genes are involved in various processes, among these are neurological signaling, the maturation of the immune system, and the inhibition of opportunistic pathogens. In this sense, it has been shown that a healthy ocular microbiota acts as a barrier against the entry of pathogens, contributing to the prevention of infections. In recent years, a relationship has been suggested between microbiota dysbiosis and the development of neurodegenerative diseases. In patients with glaucoma, it has been observed that the microbiota of the ocular surface, intraocular cavity, oral cavity, stomach, and gut differ from those observed in healthy patients, which may suggest a role in pathology development, although the evidence remains limited. The mechanisms involved in the relationship of the human microbiome and this neurodegenerative disease remain largely unknown. For this reason, the present review aims to show a broad overview of the influence of the structure and composition of the human oral and gut microbiota and relate its dysbiosis to neurodegenerative diseases, especially glaucoma.

## 1. Introduction

Glaucoma is a multifactorial neurodegenerative disorder characterized by the progressive loss of retinal ganglion cell (RGC) axons in the optic nerve head (ONH), mainly due to an increase in intraocular pressure (IOP) [1,2]. Glaucoma is the leading cause of an irreversible loss of vision, affecting over 70 million people and causing approximately 6 million cases of blindness [3,4]. Moreover, glaucoma can be broadly classified as open-angle glaucoma (OAG) and angle-closure glaucoma (ACG), whose causes can be either primary or secondary. Generally, although it is not always the case, the level of IOP is related to RGC death. IOP is mainly regulated by the fine balance of aqueous humor (AH) secretion by the ciliary body and drainage by the trabecular meshwork (TM) and uveoscleral outflow pathway. In OAG, there is an increased resistance to AH absorption by the TM; while in ACG, access to the drainage pathways is blocked [3].

OAG is the most prevalent cause of glaucoma overall, with primary OAG (POAG) being the most common type. The pathophysiology of POAG is not well understood, but the IOP increase generally seen in this disease has been associated with the reduction in the number and function of the endothelial cells in the TM. This in turn reduces AH drainage and causes ocular hypertension, which brings about biomechanical deformation of the ONH and consequently affects RGC function via axonal injury, vascular dysregulation or ischemia, oxidative stress, excitotoxicity, inflammation, autophagy, autoimmunity, and reactive gliosis [4]. In secondary OAG (SOAG), the causes of the AH outflow blockage are clear, such as in pigmentary and exfoliative glaucoma. On the other hand, regarding primary ACG (PACG), a bowing of the iris occurs due to an increased pressure variance between the anterior and posterior compartments of the eye, putting the iris in direct contact with the TM and causing a pupillary block that inhibits the AH flow. Angle crowding can also cause PACG, in which the iris is compressed against the TM and another anatomical structures such as the ciliary body [5]. Moreover, in secondary ACG (SACG), the blockage of the TM is due to complications of other diseases, such as in neovascular glaucoma [4].

Onto another topic, the human body is host to a vast variety of microorganisms, including bacteria, yeast, and viruses, which coexist in several locations of the body (e.g., the gut, skin, lung, and oral cavity) [6,7]. These microbes interact with the host in several biological processes, finally contributing to health and disease [8]. The human body’s assortment of inhabiting microorganisms has been termed the “human microbiota” and consists of 10 to 100 trillion symbiotic microbial cells held in each person [6]. On the other hand, the term “human microbiome” refers to the genes that these microorganisms contain [9].

The development of the Human Microbiome project, proposed as an initiative of the National Institutes of Health (NIH) Roadmap for Biomedical Research, made it possible to characterize the microbiome associated with various parts of the body [10]. It has also allowed us to determine if there are associations between microbiome changes and human health to evaluate the opportunities to ameliorate health through the monitoring and/or manipulation of the human microbiome [11,12]. Among the several niches where microbiota reside, of special interest is the human gut microbiota, which is considered the most important regarding health maintenance [8].

The microbiota is in constant evolution in response to host factors such as age, nutrition, lifestyle, hormonal changes, genetic background, and underlying diseases [13]. When significant changes in microbiota composition and function occur (termed dysbiosis), the host is at an increased risk for developing different conditions that can go from several gastrointestinal disorders to neurological and ophthalmological diseases, and even cancer [8,14,15,16,17,18].

The objective of this review is to present a broad panorama of the composition and inner workings of the human oral and gut microbiota and to associate its dysbiosis with human disease, with particular interest in ophthalmological conditions, especially glaucoma.

## 2. Oral and Gut Microbiota Analysis and Methods of Study

The human gut microbiota is made up of a dynamic and diverse assemblage of approximately 4 × 10^13^ bacteria of 500 to 1000 different species [7,19,20,21]. It has been reported that in the gut, the microbiota is involved in primary functions such as nutrition and metabolism of the host; immunomodulation and protection against the invasion of infectious agents or the overgrowth of resident species with pathogenic potential; and the utilization, differentiation, and maintenance of the intestinal epithelium mucosa [22,23]. Moreover, the oral cavity features one of the most diverse microbial assemblages in the human microbiome [24,25]. It has been estimated that one milliliter of saliva contains 10^8^ microbial cells, and approximately 1000 different species have been identified to be capable of oral colonization [26,27].

Onto the gut microbiota, the predominating phyla are *Firmicutes*, *Proteobacteria*, *Bacteroidetes*, *Actinobacteria*, *Fusobacteria*, *Verromicrobia*, and *Cyanobacteria* [28]. From these, *Firmicutes* and *Bacteroidetes* represent approximately 90% of the gut microbiota. The former is composed of different proportions of more than 200 different genera, such as *Lactobacillus*, *Bacillus*, *Clostridium*, *Enterococcus*, and *Ruminicoccus*, while the latter predominantly consists of the *Bacteroides* and *Prevotella* [29]. Interestingly, within the gastrointestinal tract of the same individual, there are microbiota variations depending on the anatomical part of the gut. Moreover, the human gut microbiota also varies depending on gestational age at birth, type of delivery, methods of milk feeding, human age, and antibiotic treatments [29].

Microbiota variations can also be found depending on the country of origin and ethnicity of individuals, among several other factors, meaning unique microbiota compositions may vary among populations [30]. The same can be said for the oral microbiome, which represents a special challenge since this cavity harbors habitats with unique values of oxygen concentrations, nutrient availability, temperature, exposure to immunological factors, and anatomical characteristics [25,31]. For example, soft tissues, saliva, and the tongue have a high abundance of the genus *Streptococcus*. On the other hand, in the fissures of the tongue and at the supragingival and infragingival level, the genera *Actinomyces* dominate. Other bacterial taxa such as *Veillonella parvula* and *Neisseria* can be isolated from all cavities. Likewise, bacterial assemblies made up of *Aggregatibacter actinomycetemcomitans*, *Porphyromonas gingivalis*, and *Tannerella forsythia* can perform intracellular colonization in epithelial cells of the oral cavity [32].

The gut microbiota has an essential role in human metabolism as it contains enzymes that the human genome does not encode for the breakdown of polysaccharides and polyphenols and vitamin synthesis. It also contributes to the metabolism of fatty acids, proteins, and bile acid biotransformation [33]. With this in mind, alterations in the equilibrium of the microbiota could lead to several conditions. For instance, it has been suggested that disturbances in the fecal microbial community are associated with the development of obesity, insulin resistance, and cardiovascular disease, to name a few [34]. Moreover, gut dysbiosis has also been found to trigger inflammation and immune responses that indirectly lead to carcinogenesis, especially colorectal cancer. Interestingly, respiratory diseases such as asthma and chronic obstructive pulmonary disease have also been associated to the gut microbiota via a gut–lung axis [8]. The list of gut dysbiosis associated diseases continues, and the mechanisms underlying their relation are still under investigation.

On the other hand, oral dysbiosis can locally cause dental caries and periodontal disease [35]. For instance, high sugar consumption favors the growth of aciduric microorganisms, which produce acids that demineralize the tooth enamel. *Mutans streptococci* and *lactobacilli* are the main players for dental caries generation, although other species such as *bifidobacterium*, *scardovia*, *actinomyces*, and even fungi such as *Candida albicans* have been associated with caries [36]. Moreover, in periodontitis, a pathogenic triad has been found, which includes *P. gingivalis*, *T. forsythia*, and *Treponema denticola*, although many others have been related [27]. Interestingly, the oral microbiota has not only been found to be associated with dental disease. For instance, it has been found that periodontitis is involved in the start of oral, pancreatic, genitourinary, and gastrointestinal cancers, and also with an increased risk of cardiovascular and chronic kidney disease [8]. Moreover, oral dysbiosis has also been associated with brain disorders such as Alzheimer’s disease [37,38,39].

Regarding the study of the microbiota, several methods with different purposes have been developed. These involve the fields of genomics, transcriptomics, proteomics, and metabolomics. In genomics, sequencing has been the most important method to identify the human microbiome [40,41]. There are two main techniques, 16S rRNA sequencing and metagenomic sequencing [42]. The former has been the most utilized method for microbiome research and is based on the bacterial 16S rRNA gene highly conserved sequences, which contain plenty of phylogenetic information for taxonomic classification [43]. Regarding the latter, metagenomic sequencing, also called “shotgun” sequencing, is a more complicated technique that involves the random sequencing of all the genomes of all the organisms found in a sample [44]. The sequenced DNA fragments are then compared to reference databases to identify the microorganisms but could also be assembled de novo to identify previously uncharacterized species [45]. Microbiota transcriptomics, on the other hand, provides information regarding the gene expression profiles of the microbiota, not just its genetic contents. This is achieved by the isolation and sequencing of all the mRNA sequences in a sample, later comparing the assembly obtained with reference genomes in a process known as metatranscriptomics [46]. To obtain the global analysis of the protein contents of a sample, which are dynamic, a metaproteomics approach to study the microbiota is needed. Proteomic analysis has the advantage of showing the proteins that are expressed and performing at a specific time point. Generally, mass spectrometry-based methods are used, which serve to identify biomarkers and pathways present in the microbial populations [47]. Finally, in metabolomics, the metabolites produced by the microbiota are studied, giving insights into the processes occurring in response to stimuli or interactions. Mass-spectrometry-based assays are also usually employed in metabolomic studies [48].

## 3. Oral and Gut Microbiome in Glaucomatous Neurodegeneration

### 3.1. General Pathway

In order to properly understand how a series of changes in gut and oral microbiota lead to the neurodegenerative changes observed in glaucoma, it is sensible to start at the end of the physiopathological path and work backwards. As previously stated in the introduction, ocular hypertension due to increased IOP is a common endpoint for most cases of glaucoma, be it open-angle or closed-angle. Among the wide array of reasons that can trigger such an ocular environment of elevated pressure, a state of systemic inflammation and the circulation of vasoactive and reactive oxygen species could contribute to the initial damage that eventually causes glaucoma downstream.

A state of chronic low-grade systemic inflammation and endotoxemia can be explained by various concurrent events. On the one hand, alterations in body mass index (BMI), obesity, and fat storage have previously been explored and observed to be associated with the development of increased IOP. Inflammatory strain, adipose-derived metabolic dysregulation, and neural deterioration lead to a series of physiopathological processes such as increased circulating proinflammatory cytokines and interleukins (e.g., INF-α and IL-6). Various studies have pointed out that obesity has a strong connection with other neurodegenerative disorders such as Alzheimer’s disease [49].

On the other hand, the aforementioned systemic effects can be caused by a state of dysbiosis as well. Intestinal equilibrium and the control of inflammation (i.e., suppresion) rely on the constantly evolving relationship between the gut microbiota and the immune system of the host. Acting as a physiological and biochemical barrier, the intestinal epithelium prevents the entry of foreign antigens, including those from food, infections, and toxins. Beneficial bacteria within the gut microbiome safeguard the host from pathogens through diverse methods, including outcompeting for nutrients, changing environmental factors, influencing the development of immune cells (known as immune-mediated resistance), and generating metabolites that hinder growth or have bactericidal properties [50].

**Figure 1:**
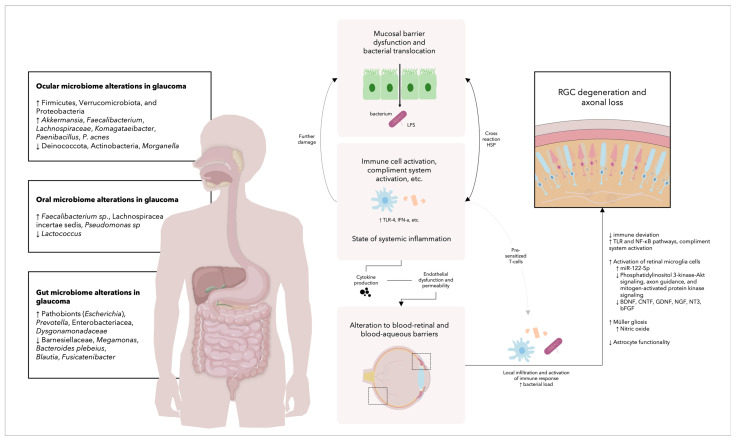
Overview of the relationship between microbiome alterations and glaucoma development. In individuals diagnosed with glaucoma, there is a noticeable shift in the relative abundance of various microorganisms within specific sites, including the ocular, oral, and gut microenvironments. The influence of any single bacterium is overshadowed by the overall collective presence of the microbial population. Localized inflammation triggered by this bacterial presence, alongside additional factors such as adipose tissue levels, dietary habits, and age, contributes to the disruption of cellular barriers and tight junctions. Consequently, bacteria breach these constraints, prompting the activation of the immune system. This immune response involves the mobilization of immune cells and the complement system in reaction to bacterial components such as lipopolysaccharides, culminating in systemic inflammation. This inflammatory process is known to compromise the integrity of the blood–retinal and blood–aqueous barriers, allowing the invasion of both bacteria and immunological constituents, including T-cells previously sensitized to bacterial elements such as heat shock proteins, which exhibit molecular mimicry with host antigens. Subsequent immunological activation within the ocular environment exceeds its innate regulatory capacity, fostering various pathological alterations that ultimately lead to the degeneration of retinal ganglion cells and loss of axons. BDNF, brain-derived neurotrophic factor; bFGF; basic fibroblast growth factor; CNTF, ciliary neurotrophic factor; GDNF, glial cell line-derived neurotrophic factor; HSP, heat shock proteins; IFN-a, interferon alpha; LPS, lipopolysaccharides; miR, microRNA; NF-κB, nuclear factor kappa-light-chain-enhancer of activated B cells; NGF, nerve growth factor; NT3, neurotrophin-3; RGC, retinal ganglion cell; TLR-4, Toll-like receptor 4; NF-κB.

These two inciting incidents, fat-related changes and gut endotoxemia, do not exist as individual physiopathological pathways; instead, they share extensive overlap. A state of mucosal barrier dysfunction (especially due to the disruption of tight junctions) often leads to enhanced intestinal permeability and what is known as leaky gut syndrome. The translocation of commensal microbes into the body activates several immunological pathways, such as Toll-like receptors (TLRs) and NOD-like receptors (NLRs), which are among the main families that comprise the innate immunity pattern recognition receptors (PRRs) superfamily [51]. Particularly, these receptors interact with the bacterium’s lipopolysaccharides (LPS) (a fundamental component of Gram-negative bacterias’ outer membrane), which traverse through the gut epithelium directly or by infiltrating chylomicrons. An increased amount of circulating free fatty acids (FAAs), observed in states of increased human adipose content, can also concomitantly stimulate the expression of certain TLRs, most importantly TLR4, on macrophages, adipocytes, and adipose tissue. These interactions among pathogen-associated and damage-associated molecular pathways with TLRs and NLRs, therefore, cause the release of several inflammatory mediators such as the previously mentioned IL-6 and TNF-α, as well as increased NF-κB activity with the transcription of IL-1β and IL-18 [52]. An overview of the relationship between microbiome alterations and glaucoma development can be seen in Figure 1.

#### 3.1.1. Oral Microbiota

In addition to the previously stated, the oral microbiome contributes to glaucoma pathophysiology as well. The mechanism by which increased bacterial loads can lead to neurodegeneration is quite similar to gut dysbiosis, for example, by the activation of microglia in the optic nerve and retina, mediated through TLR4 signaling and complement upregulation [37]. Some studies have observed reduced *Lactococcus* loads in glaucoma patients’ oral microbiome, which suggests that their absence in the microbiota could play a role in the pathophysiology of glaucoma and can perhaps even be used as a surrogate marker for this condition. The deleterious effects due to their absence may be due to the lack of competition with other taxa in the microenvironment or due to the decreased antimicrobial activity of H_2_O_2_ produced by downregulation of the lactic acid bacteria [16]. Bacteria which have been observed to increase in some studies, albeit in uveitic glaucoma, include *Faecalibacterium* sp., *Lachnospiracea incertae sedis*, and *Pseudomonas* sp. [53].

#### 3.1.2. Gut Microbiota

The synthesis of data from both the MetaHit and the Human Microbiome Project has offered the most extensive understanding of the microbial diversity associated with humans thus far. Through these studies, 2172 species have been isolated in humans, categorized into 12 distinct phyla, with the majority (93.5%) falling into *Firmicutes*, *Proteobacteria*, *Bacteroidetes*, and *Actinobacteria* [21]. These bacteria are subject to various alterations due to disease, antibiotic use, host diet, culture, etc., including aging-related changes that encompass the reduction in core commensal taxa (such as *Prevotella*, *Firmicutes*, and *Bifidobacterium*) and expansion of pathobionts (such as *Escherichia*, *Parabacteroides*, *Ruminococcaceae*, and *Streptococcus*) and a second group of commensals (such as *Akkermansia*, *Odoribacter*, *Butyricimonas*, *Oscillospira*, *Christensenellaceae*, and *Barnesiellaceae*) [54]. These alterations may explain low systemic inflammatory status, malnutrition, gut tight-junction alterations, increase in risk for obesity, diabetes, etc.

### 3.2. Pathway in Glaucoma

Having explored these systemic effects in previous paragraphs, as well as changes in gut and oral microbiomes, they can now be followed through to their natural conclusion in ocular pathology. It should be pointed out that neuronal loss due to infectious/inflammatory processes in the periphery is not specific to glaucoma, as we have previously pointed out in neurodegenerative diseases such as Alzheimer’s and Parkinson’s disease. What seems to be the case is that chronic peripheral inflammation due to chronic exposure to microbial products caused by disturbances of the local microbiome in the oral and gut cavity can lead to the progression of neurodegeneration that genetic, epigenetic, or environmental factors have initiated. Therefore, what matters more is perhaps not the particulars of one specific bacterium, but rather the load of bacteria and the balance between beneficial and malicious bacteria overall; this, in turn, may account in part for the chronicity and periodic exacerbations that are typical in the course of neurodegenerative conditions [53].

Typically, the eye is acknowledged as a site with immune privilege compared to the rest of the body. This immune regulation within the eye involves active local immunological suppression, facilitated in part by the blood–aqueous and blood–retina barriers (BRB), as well as the local production of immunosuppressive cytokines and neuropeptides. In cases of infection or injury prompting immune cell infiltration into the eye, these cells are often prompted to undergo apoptosis through the activation of Fas-FasL signaling, without triggering inflammation or tissue harm. When antigenic material is introduced into the eye, it triggers immune deviation or suppression of T-cell-mediated immunity, resulting in peripheral immune tolerance to the antigens. This mechanism is referred to as anterior chamber associated immune deviation [55,56].

The unregulated tilt towards a systemic proinflammatory state has been observed in studies to alter the barrier functions of both the blood–brain barrier and the BRB, which then allows the passage of immune cells, such as macrophages and activated T cells, as well as microbial toxins and metabolites to cross into these immune-privileged sites. This deduction of evident innate and adaptive immunity activation is supported by the observed presence of elevated levels of complement components, autoantibodies, and various inflammatory markers in the aqueous humor and vitreous of individuals suffering from glaucoma. Moreover, a study observed that the induction of germ-free mice (i.e., with an absence of gastrointestinal bacteria) abolished the development of glaucoma by reducing human/bacterial heat shock proteins cross-reacting glaucomatous T-cell responses, providing evidence that glaucomatous neurodegeneration is partly facilitated by T cells that have been presenstitized through exposure to commensal microflora [57].

A study found that glaucoma patients exhibited a distinctive composition in their gut microbiome, characterized by an abundance of *Dysgonamonadaceae* and a reduced presence of *Barnesiellaceae*, as determined through metagenomic sequencing. This particular microbiome profile was associated with the heightened production of short-chain fatty acids (SCFAs) in both fecal and blood samples. Significantly reducing the levels of these glaucoma-associated gut microbiome bacteria led to a notable decrease in retinal ganglion cell loss by mitigating the activation of retinal microglia cells and the excessive production of inflammatory cytokines in an acute glaucoma mouse model. Conversely, treatment with SCFAs exacerbated microglia activation. Mechanistically, diminishing the glaucoma-specific gut microbiome bacteria altered the retinal microRNA profile, including miR-122-5p, which negatively regulates focal adhesion, tight junctions, and TGF-ß signaling, consequently resulting in neuroprotection through the inhibition of retinal inflammation. These findings were corroborated by demonstrating that fecal microbiome transplantation from glaucoma patients significantly intensified retinal microglia activation and heightened retinal inflammation [58,59].

Further studies suggest that the gut–retinal axis might influence the activation of retinal microglia via changes in retinal miRNA expression. Administering antibiotic cocktails to mice notably reduced the overall density of the gut microbiome, resulting in decreased activation of retinal microglia and reduced production of pro-inflammatory cytokines in the retina. This, in turn, led to the neuroprotection of retinal cells due to alterations in miRNA expression within the neuroretina. Specifically, 26 miRNAs were found to be downregulated, including MiR-122-5p, while 8 miRNAs were upregulated. These differentially expressed miRNAs were associated with pathways such as phosphatidylinositol 3-kinase-Akt signaling, axon guidance, and mitogen-activated protein kinase signaling [59,60].

Various changes occur due to inflammation in the ocular microenvironment. From one pathway, a sustained and cumulative microenvironment of stress in the eye induces a loss of local protective and/or anti-inflammatory responses, which are typically undertaken by microglia (as previously explored) and astroglia, that consists of Müller cells and astrocytes that provide metabolic support to neurons, neurological regulation of ionic concentrations, and neuroprotective activities [61,62].

Expanding upon these pro-neurological activities, when in a non-inflammatory state, microglia secrete brain-derived neurotrophic factor (BDNF), ciliary neurotrophic factor (CNTF), glial cell line-derived neurotrophic factor (GDNF), nerve growth factor (NGF), neurotrophin-3 (NT3), and basic fibroblast growth factor (bFGF), activities which are disrupted by activation. Overactivation of microglia, due to direct phagocytosis or cytokine stimulation, results in the production of more proinflammatory cytokines and increased oxidation and nitrification reactions, thereby endangering the retinal neurons; this conclusion is supported by the fact that the suppression of microglial activation with minocycline protected RGCs from elevated IOP-induced cell death in rodents [63,64].

Müller cells play a crucial role in regulating and breaking down substances like glutamate and GABA, while also secreting the antioxidant glutathione. Müller gliosis, marked by the heightened expression of glial fibrillary acidic protein and activation of extracellular signal-regulated kinases, is observed in various retinal diseases. Initially, following injury, Müller gliosis might offer neuroprotection through the generation and release of antioxidants and trophic factors, including the expression of CNTF. However, advanced stages of gliosis have been linked to cell death and the formation of a glial scar, which hinders neuronal regeneration. A notable pathological aspect of gliosis is the buildup of nitric oxide, which has been demonstrated to induce intracellular damage by impeding mitochondrial function, reducing ATP levels, and causing direct harm to DNA [65].

Among their distinct functions, astrocytes maintain the structure of the environment by secreting extracellular matrix molecules and by facilitating myelination and myelin maintenance by clearing extracellular ions and neurotransmitters, as well as secreting pro-myelinating factors [66]. The functionality of astrocytes surrounding the lamina cribrosa diminishes, leading to pathological alterations in the extracellular matrix characterized by abnormal deposits. These deposits are located within laminar pores formerly occupied by clusters of RGC axons. Clinically, these changes manifest as optic cupping when assessing the cup-to-disc ratio [67].

All these cells adopt a phenotypic change and activation, with detrimental effects such as the activation of TLR and NF-κB pathways, activation of the complement system, and abnormal cell migration and infiltration patterns. These processes lead to direct cytotoxicity, abnormal regulation of protective axonal receptor expression, and necroptosis, to name a few. This induced damage to the RGCs and other structures leads to the ocular alterations that eventually increase IOP, which causes further activation of inflammatory cells, perpetuating a cycle of ever-increasing dysfunction and deterioration [68].

Delving into specific details, hub genes such as NFKB1, IL18, TLR9, FKBP2, KITLG, and HDAC4 have been identified in patients with POAG and in databases of gut microbiota regulation, influencing the NF-κB and mitogen-activated protein kinase (MAPK) signaling pathways. These genes exhibit significant correlation with the regulation and activation of leukocytes, particularly macrophages. Consequently, these six hub genes are likely to play crucial roles by impacting macrophage activity during the progression of POAG and in the regulation of gut microbiota [69,70].

### 3.3. Alterations in Normal Ocular Microbiota

To give closure to this section, it should be mentioned that although it is an immunologically privileged site, impressively, the eye is still populated by its own microbiome of sorts. Studies have found that the ocular surface of normal individuals prevailed with *Actinobacteria* (79.5%) and *Firmicutes* (12.9%), while Gram-positive bacteria such as *Corynebacterium* (71.7%), *Cutibacterium* (5.4%), and *Blautia* (4.4%) were the most dominant genus. In contrast, the surface of glaucoma eyes had more *Firmicutes*, *Verrucomicrobiota*, and *Proteobacteria* but fewer *Deinococcota* and *Actinobacteria*. Further analysis found glaucoma eyes to have more anaerobic, Gram-negative bacteria such as *Akkermansia*, *Faecalibacterium*, *Lachnospiraceae*, and *Komagataeibacter* [54,71]. A list of the identified microbes which could be associated with glaucoma pathogenesis are found in Table 1.

## 4. Impact of Dysbiosis in Other Neurodegenerative Ocular Diseases

### 4.1. Age Related Macular Degeneration

Age-related macular degeneration (AMD) is the leading cause of legal blindness in the industrialized world. AMD is characterized by the accumulation of extracellular deposits, namely drusen, along with progressive degeneration of photoreceptors and adjacent tissues [77]. Advanced AMD is classified into the nonexudative or atrophic form (dry AMD) and the exudative or neovascular form (wet AMD). Multiple genetic factors, lipid metabolism, oxidative stress, and aging play a role in the etiology of AMD [78].

The relation between gut health and AMD has been well explored for many years. Oral supplementation with the Age-Related Eye Disease Study (AREDS) formulation (antioxidant vitamins C and E, beta carotene, and zinc) has been shown to reduce the risk of progression to advanced AMD since 1999, for example [79]. Dysregulated immune activation involving the infiltration of microglia and macrophages into the subretinal and choroidal regions, the activation of mast cells, and immune activation of the retinal pigment epithelium (RPE) are all presumed to contribute to the development or advancement of AMD. Nevertheless, the source of this inflammation remains unclear [80].

In a clinical case–control study comparing the microbiomes of individuals with advanced AMD to control patients, it was observed that AMD patients had elevated levels of *Prevotella*, *Holdemanella*, and *Desulfovibrio*. Notably, several of these species have been linked to other inflammatory conditions. Furthermore, analysis of gut metagenomes from neovascular AMD patients and controls revealed an increase in *Anaerotruncus*, *Oscillibacter*, *Eubacterium ventriosum*, and *Ruminococcus* torques, alongside a decrease in Bacteroides eggerthii among AMD patients [80,81].

### 4.2. Diabetic Retinopathy

Diabetic retinopathy (DR), a severe and vision-threatening condition, stands as one of the most prevalent complications specific to diabetes. It is now understood that DR is an inflammatory and neurovascular complication, with neuronal impairment or dysfunction preceding clinical microvascular damage. The pathophysiological processes that harm pancreatic β-cells—such as inflammation, insulin resistance, epigenetic alterations, fuel excess, and an abnormal metabolic environment—likewise contribute to cellular and tissue injury, resulting in organ dysfunction and heightening the risk of all complications, including DR [82].

In clinical terms, DR is categorized into two stages: non-proliferative diabetic retinopathy (NPDR) and proliferative diabetic retinopathy (PDR). NPDR constitutes the initial/early phase of DR, marked by heightened vascular permeability and capillary occlusion in the retinal vasculature. During this stage, retinal abnormalities such as hemorrhages, microaneurysms, and hard exudates can be identified through fundus photography, although patients may not experience symptoms. PDR, an advanced stage of DR, is distinguished by the development of neovascularization. In this phase, patients may encounter significant vision impairment due to bleeding from the newly formed abnormal vessels into the vitreous (vitreous hemorrhage) or the presence of tractional retinal detachment. Diabetic macular edema (DME) can manifest at any stage of DR and is characterized by swelling or thickening of the macula caused by the accumulation of fluid, both beneath and within the retina, triggered by the breakdown of the blood–retinal barrier [83,84].

As with other diseases mentioned in this review, studies have established the compositional alteration of gut microbiota in diabetes and diabetic retinopathy. Studies have reported *Blautia* as the most abundant genus; in addition, increased levels of *Bifidobacterium* and *Lactobacillus* and decreased levels of *Escherichia-Shigella*, *Faecalibacterium*, the *Eubacterium hallii* group, and *Clostridium* genera were observed in diabetic patients over healthy individuals. It should be mentioned that *Pasteurellaceae* was found to be a differentiator in patients with only diabetes (increased) and patients with DR (decreased) in comparison with healthy individuals [85].

### 4.3. Stargardt

Stargardt disease (STGD1) is an inherited retinal dystrophy caused by mutations in ABCA4, following an autosomal recessive pattern. It is identified by the accumulation of lipofuscin-like materials beneath the retina and results in bilateral and centrifugal vision loss. Despite considerable advancements in comprehending STGD1, there are currently no approved treatments available [86].

The use of carotenoid supplementation, serving as a source of retinoids, has been proposed as a potential dietary strategy to enhance outcomes in STGD. This approach aims to protect the macula, as STGD patients have been observed to have lower serum levels of carotenoids compared to healthy controls. While carotenoid supplementation has shown positive effects on visual performance in ABCA4-knockout mice, no clinical trials involving nutritional interventions have been conducted thus far. In fact, there are contradictory findings regarding the impact of dietary vitamin A on STGD outcomes, with some laboratory research indicating that vitamin A supplementation may lead to an increased accumulation of lipofuscin (albeit without significant effects on retinal function), and thus, recommend that STGD patients avoid retinoid supplements [87,88].

## 5. Microbiome-Based Therapeutics in Glaucoma

The gut microbial pattern of glaucoma patients has been demonstrated to enhance the generation of SCFAs in both fecal and blood samples [89]. Decreasing the presence of these specific gut microbiome bacteria associated with glaucoma significantly mitigated the loss of RGC by alleviating the activation of retinal microglia cells and the excessive production of inflammatory cytokines in an acute glaucoma mouse model [59]. Conversely, treatment with SCFAs exacerbated the activation of microglia [54].

Mechanistically, the reduction in glaucoma-associated gut microbiome bacteria resulted in alterations to the retinal microRNA profile, including the downregulation of MiR-122-5p, ultimately providing neuroprotection by inhibiting retinal inflammation [59].

- Diet

In a study, a link between following the Mediterranean-DASH Intervention for Neurodegenerative Delay (MIND) diet and a decreased occurrence of OAG was notably stronger compared to adhering to either the Mediterranean diet or the Dutch dietary guidelines [90]. These authors found that the MIND diet did not show any association with IOP, suggesting that its potential protective impact on OAG likely involves preserving retinal ganglion cells rather than reducing IOP.

Numerous researchers have provided evidence indicating that increasing the intake of nutrients such as B vitamins, omega-3 fatty acids, and nitrates, along with incorporating specific foods like green leafy vegetables and fruits into one’s diet correlates with a decreased likelihood of developing glaucoma. Essential components contributing to this connection encompass a heightened consumption of green leafy vegetables, berries, and fish [91,92].

- Fecal microbiota transplantation

Fecal microbiota transplantation (FMT) involves transferring fecal material from a healthy donor into the gastrointestinal tract of patients to restore their dysbiotic microbiota both in composition and function [93]. Once the role of fecal microbiota in glaucoma development is established, it would be beneficial to explore its relationship with fecal transplantation to manipulate the microbiome and assess its potential benefits [17,94].

- Antibiotics

The presence of an active gastric *Helicobacter pylori* infection has been correlated with the occurrence of glaucoma [90,95]. The association between the mentioned pathogen and its effects initiates an increased likelihood of developing chronic atrophic gastritis. This condition leads to the accumulation of homocysteine due to diminished absorption of vitamin B-12 and folate. Subsequently, hyperhomocysteinemia triggers vascular endothelial dysfunction, which is associated with POAG and SOAG [73,96].

Moreover, chronic *H. pylori* infection stimulates the increased secretion of inflammatory factors, resulting in heightened permeability of the BRB. This heightened permeability promotes the apoptotic death of RGCs and induces constriction of the anterior vessels of the optic nerve. Consequently, the eradication of *H. pylori* through therapeutic interventions, such as omeprazole, clarithromycin, amoxicillin, or other antibiotic regimens, holds potential benefits for patients with glaucoma [73]. This association suggests that addressing the bacterial infection in the gastric system may positively influence the management or progression of glaucomatous conditions. Further research into the specific mechanisms underlying this relationship and the efficacy of various eradication protocols is warranted to optimize treatment strategies for affected individuals.

## 6. Future and Perspective

Considering the importance of immunological pathways within the pathophysiology of glaucoma, it is reasonable to assume that immunomodulation might be a viable point of convergence for therapeutic efforts. The issue with these therapies is the dangerous profile of side effects that they induce in patients; therefore, perhaps what is worth exploring is the identification of triggers that cause immune exacerbation and an inflammatory state and seek to eradicate these sources. Although its role or impact as a primary source of treatment is questionable, it is conceivable that microbiome modulation may contribute to the prevention of diseases such as glaucoma, as well as serve as an adjuvant to other therapies. For example, various studies have stated that there is a statistically significant association between *H. pylori* infection and POAG, as well as with other subtypes. Although the eradication of *H. pylori* will not cure every case of glaucoma, perhaps there would be a diminishment in incidence [97].

Key modifiers in reducing a proinflammatory state include the following: dietary adjustments with nutrition optimization, exercise and increased activity, as well as the use of probiotics. Utilizing the latter approach could enhance the diversity of gut microbiota, stimulate mucus production, and safeguard tight-junction proteins by reducing the presence of LPSs. Additionally, it may reduce the emergence of inflammatory biomarkers and dampen unnecessary activation of the immune system. In vitro and in vivo studies have demonstrated that the most used probiotics in humans (e.g., *Lactobacillus*, *Bacillus*, *Saccharomyces*, and *Bifidobacterium*), modulate cytokine production by immune cells, as well as tolerance acquisition induction [98].

During in vitro studies examining the effects of *Lactobacillus* and *Bifidobacterium* on Parkinson’s disease, it was observed that the expression levels of the pro-inflammatory cytokines IL-1, IL-8, and TNF-α were decreased [99]. Conversely, there was an increase in the expression of the anti-inflammatory regulator TNF-β [100,101]. These changes resulted in an improvement in motor signs and symptoms in the group treated with probiotics. Effects such as these should provide the motivation and groundwork to further explore therapies that resolve dysbiosis.

## 7. Conclusions

In conclusion, dysbiosis in the oral and gut microbiome represents a potential factor involved in the pathogenesis of glaucoma. These microbiota variations generate metabolic problems, toxic products, decreased defenses, and autoimmune problems. Despite this, the underlying mechanisms involved in this dysbiosis and its influence on neurodegenerative diseases, like glaucoma, are still unclear. The use of 16S rRNA gene sequencing is a useful tool that allows the identification of bacterial species involved in pathogenicity or host benefit. Therefore, the characterization of this altered microbiota and its influence on ocular diseases, especially glaucoma, is relevant to deepen current knowledge of this pathogenic pathway, as well as for the therapeutic development of autoimmune and inflammatory diseases.

## Figures and Tables

**Table 1:** Current evidence of microbiome alterations related to glaucoma.

Microbe	Location	Alteration in Glaucoma	Ref.
*Paenibacillus*	Ocular	Increase	[53]
*Firmicutes*	Ocular	Increase	[71]
*Verrucomicrobiota*	Ocular	Increase	[71]
*Proteobacteria*	Ocular	Increase	[71]
*Lachnospiraceae*	Ocular	Increase	[71]
*Komagateiabacter*	Ocular	Increase	[71]
*Morganella*	Ocular	Decrease	[53]
*Deinococcota*	Ocular	Decrease	[71]
*Actinobacteria*	Ocular	Decrease	[71]
*Faecalibacterium*	Ocular/Oral	Increase	[53,71]
*Lactococcus*	Ocular/Oral	Decrease	[53]
*Akkermansia*	Ocular/Gut	Increase	[71]
*Propionibacterium Acnes*	Intraocular	Increase	[72]
*Lachnospiracea incertae sedis*	Oral	Increase	[53]
*Pseudomonas sp*	Oral	Increase	[53]
*Helicobacter pylori*	Oral/Gut	Increase	[73,74]
*Escherichia coli*	Gut	Increase	[75]
*Prevotella*	Gut	Increase	[75]
*Enterobacteriaceae*	Gut	Increase	[75]
*Dysgonamonadaceae*	Gut	Increase	[59]
*Barnesiellaceae*	Gut	Decrease	[59]
*Megamonas*	Gut	Decrease	[75]
*Bacteroides plebius*	Gut	Decrease	[75]
*Blautia*	Gut	Decrease	[76]
*Fusicatenibacter*	Gut	Decrease	[76]
*Bifidobactierum adolescentis*	Gut	Not well defined. May regulate macrophage activity associated with glaucoma.	[70]
*Lactobacillus paracasei*	Gut	Not well defined. May regulate macrophage activity associated with glaucoma.	[70]

Current evidence of microbiota alterations related to glaucoma.

## Data Availability

Not applicable.
